# A deep learning framework for remaining useful life prediction of turbofan engines with partial sensor failure

**DOI:** 10.1371/journal.pone.0347312

**Published:** 2026-04-29

**Authors:** Dongdong Tang

**Affiliations:** Suining Branch, Civil Aviation Flight University of China, Suining, Sichuan, China; Sunway University, MALAYSIA

## Abstract

During long-term operation, turbofan engine sensors often suffer from partial damage or signal loss caused by complex flight environments, harsh mechanical vibrations, and thermal stresses. Such degradation in sensor reliability leads to incomplete or inaccurate monitoring data, which significantly reduces the precision of remaining useful life (RUL) prediction and poses potential risks to the safety and maintenance scheduling of aero-engines. To address this challenge, this paper proposes a novel generative regression model based on Long Short Term Memory Generative Adversarial Network to achieve robust life prediction under sensor damage conditions. The proposed model first employs a missing-parameter generator to fill in the lost sensor data, which helps restore the integrity of feature inputs. Then, we use an RUL predictor to extract the temporal degradation information from the reconstructed features for more accurate RUL estimation. Experiments conducted on the NASA C-MAPSS dataset, a widely used benchmark for turbofan engine degradation analysis, demonstrate that the proposed model maintains high prediction accuracy even under partial sensor failure scenarios and outperforms multiple baseline methods. The results verify the robustness, generalization, and reliability of the model under complex operating conditions. This study provides a task-oriented and unified modeling approach to improving turbofan engine health management, offering valuable guidance for enhancing system safety, reliability, and predictive maintenance efficiency.

## Introduction

As the power core of modern aircraft, the operational health of aero-engine is directly related to flight safety, operational efficiency and economic cost [1,2]. Because of its excellent comprehensive performance, turbofan engine has become the core component of modern aviation propulsion system [3]. However, as a complex system operating in extreme high temperature, high pressure and high speed environment over prolonged periods, the performance of each component will inevitably degrade with the service time, and eventually lead to functional failure [4,5]. Therefore, accurate Remaining Useful Life (RUL) prediction of turbofan engines has become the core task of predictive health management, which is of vital significance for ensuring flight safety, optimizing maintenance strategies, and reducing life-cycle costs [6].

In recent years, with the rapid development of sensing technology and big data analysis capabilities, data-driven methods, especially deep learning technology, have achieved remarkable results in the field of RUL prediction [7,8]. These methods are capable of automatically learn complex nonlinear degradation laws from historical engine operation monitoring data, thereby avoiding the limitations of traditional physical model, such as high modeling complexity and narrow applicability [8]. Among them, recurrent neural network architectures such as long short-term memory networks, which can effectively capture time series dependencies, have shown better prediction performance than traditional methods [6]. More recently, advanced deep learning architectures such as attention-based networks and Transformer variants have been widely explored to further improve long-term degradation modeling capability. For example, several recent studies have introduced probabilistic modeling and adversarial learning mechanisms into Transformer-based RUL prediction frameworks, such as Bayesian adversarial Transformer models and adversarial adaptation networks with feature disentanglement, which aim to improve prediction stability, uncertainty modeling, and cross-domain generalization under complex operating conditions [9,10]. These developments demonstrate the growing interest in designing more powerful data-driven architectures for industrial prognostics. However, a fundamental challenge that is often overlooked in the ideal experimental settings, yet unavoidable in real-world engineering practice, lies in the reliability of sensor measurements, which serve as the primary data source for these predictive models.

In the actual harsh operating environment, the turbofan engine sensors inevitably continue to bear the test of complex flight conditions, continuous severe mechanical vibration and transient thermal shock, which easily leads to partial sensor damage or intermittent signal loss [11]. This kind of “partial sensor failure” is different from complete failure, and its manifestation is more hidden and complex, which may cause data missing at random, accuracy degradation, or systematic bias. The resulting incompleteness and inaccuracy of the data seriously destroy the integrity and authenticity of the engine health status characteristics. When these contaminated low-quality data are directly input into the prediction model trained on the assumption of complete “clean” data, a series of chain reactions such as feature distortion, error propagation and accumulation will be caused, resulting in a decline in the prediction accuracy of the model.

Although deep learning has made significant progress in RUL prediction [12,13], traditional RUL prediction methods still lack a robust processing mechanism for missing data when facing special situations such as missing or abnormal sensor data due to faults. Most studies often rely on the model structure based on data integrity in the training and inference stage, which implicitly assumes that the input data is relatively complete or only subject to small noise interference. Therefore, in the scenario of high proportion of missing or systematic failure, RUL cannot be robustly predicted accurately, and the performance is likely to be greatly reduced. In addition, existing deep learning models usually try to preprocess missing data through data imputation (e.g., linear interpolation, KNN interpolation) [14]. However, such methods aim to optimize the reconstruction error or the fidelity of the observed distribution, which is usually independent of downstream prediction tasks. This decoupled process may result in repaired data, although statistically reasonable, missing key discriminative features that are most relevant to the device degradation process, thus limiting the upper bound of the final prediction performance.

Recently, studies in intelligent fault diagnosis have explored various hybrid or fusion-based deep learning strategies, where generative models, feature extractors, and classification networks are combined to handle complex and noisy industrial signals [15]. For example, combinations of autoencoders and recurrent neural networks (e.g., SDAE–GRU models) [16], generative adversarial networks integrated with convolutional neural networks or capsule networks [17], as well as hybrid data fusion frameworks based on deep belief networks [18] have been used for signal reconstruction, feature learning, and fault classification in mechanical systems. However, most of these approaches adopt a modular or sequential design, where data reconstruction and downstream prediction are treated as independent processes, rather than being jointly optimized in a task-driven manner. In contrast to these approaches that treat data repair and prediction as two separate stages, a unified learning framework that tightly couples data reconstruction with degradation prediction, and explicitly incorporates task-oriented constraints into the reconstruction process, may better preserve degradation-sensitive features and improve robustness under missing data conditions. The decrease in prediction accuracy and increase in uncertainty caused by the above drawbacks not only affect the optimization of maintenance strategies, but also may bring safety risks, especially in high-risk flight missions and long-period operation conditions.

**Fig 1:**
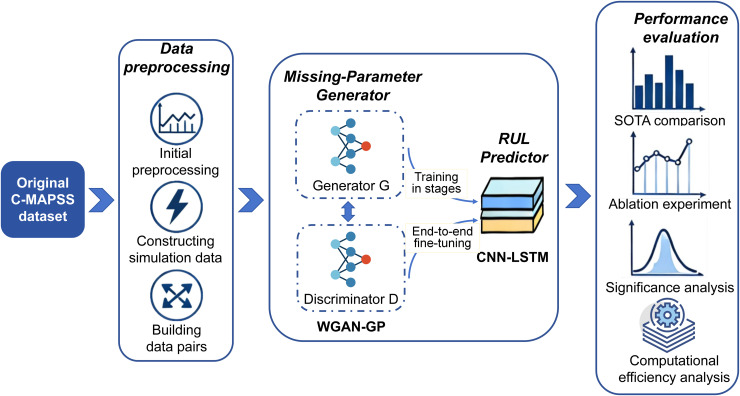
End-to-end workflow of the proposed generative repair and RUL prediction framework under sensor failures. The framework starts from the original dataset and proceeds through data preprocessing, including initial preprocessing, sensor fault simulation, and dataset pair construction. The corrupted sensor sequences are then repaired by the missing-parameter generator based on the WGAN-GP architecture, followed by RUL estimation using a CNN–LSTM predictor.

In order to solve the above problems, this paper proposes a novel robust Generative regression framework based on Long Short-Term Memory Generative Adversarial Network (see Fig 1). It realizes the end-to-end robust RUL prediction under the condition of partial sensor failure. The framework consists of two core components: the first is the Missing-Parameter Generator, which uses a WGAN-GP based generative network to reconstruct the lost signal under the premise of considering the time dependence and the correlation between sensors. The second is RUL Predictor, which takes the complete reconstructed feature sequence as input and extracts degradation patterns and performs RUL estimation based on CNN-LSTM deep temporal regression model. The core contributions of this paper can be summarized as follows:

1. A novel robust RUL regression prediction method is proposed for partial sensor failures. The proposed method tightly couples the task-oriented data repair and the final RUL prediction into a unified, collaborative optimization learning framework, enabling robust recovery and effective utilization of degradation-related information under missing data conditions, and distinguishing it from existing hybrid or fusion-based approaches that mainly rely on modular combinations of models.
2. A hybrid optimization objective combining adversarial loss and regression loss is designed. Through this design, the discriminator not only needs to distinguish the authenticity of the data, but also indirectly evaluates the “utility” of the generated data for the prediction task, thereby guiding the missing-parameter generator to preserve and enhance degradation relevant features during reconstruction, enabling task-driven reconstruction rather than conventional distribution-driven imputation, and shifting the paradigm from “blind filling” to “task-oriented intelligent repair”.
3. Experiments systematically evaluate the generation and regression performance of the proposed model on the NASA C-MAPSS benchmark dataset. The results show that the proposed framework can maintain high prediction accuracy even in the case of partial sensor failures, and outperforms the traditional baseline methods in a number of performance indicators. This confirms the robustness and superiority of the proposed method, highlighting the effectiveness of unified modeling for RUL prediction under incomplete sensing conditions and its practical value for reliable predictive maintenance in real-world industrial environments.

Unlike conventional RUL prediction approaches that treat data imputation and prognostic modeling as separate stages, the proposed hybrid generative–regression framework integrates task-oriented signal reconstruction with RUL prediction through joint optimization of adversarial and regression objectives, enabling the preservation of degradation-sensitive features and improving robustness under partial sensor failure conditions.

## Related work

### RUL prediction based on deep learning

As a key part of Prognostics and Health Management (PHM), RUL prediction plays a critical role in ensuring the safe operation of aero-engines and reduce maintenance costs. With the development of sensor technologies and big data analytics, RUL prediction methods based on deep learning have gradually become a major research focus. Compared with traditional physics-based methods, deep learning can automatically learn degradation features from massive monitoring data, establish a mapping relationship between data and RUL, and show strong end-to-end learning ability [12].

Early studies mainly used basic network structures such as convolutional Neural Network (CNN) and Recurrent Neural Network (RNN) for RUL prediction. Li et al. [19,20] proposed a deep CNN-based model to predict RUL, which can automatically learn representative features from raw sensor data and avoid the tedious manual feature selection process in traditional methods. However, researchers quickly found that a single CNN has limitations when dealing with time series data, since RUL prediction is inherently a time-dependent problem. To this end, A. Z. Hinchi and M. Tkiouat [21] combined CNN with Long Short-Term Memory Network (LSTM), which used CNN to extract spatial features and LSTM to capture temporal dependencies, forming a widely adopted CNN-LSTM hybrid architecture. This two-channel model structure was later adopted and improved by many researchers [22–24].

To further improve the modeling of long-term dependence of time series, bidirectional recurrent neural network and attention mechanism have been introduced into the field of RUL prediction. R. Wu and J. Ma [25] proposed a prediction model based on Bi-LSTM (Bidirectional long Short-Term memory network), which can learn time information from both front and back directions and make full use of context information in the sequence. Subsequently, researchers found that relying solely on the RNN structure still suffers from low computational efficiency and limited capability in capturing long-range dependency. On this basis, to enhance the model’s ability to focus on critical degradation stages and to improve prediction reliability, attention mechanisms have been introduced into the fields of RUL prediction and fault diagnosis. Attention-enhanced models based on CNN-BiLSTM have demonstrated notable advantages in time-series prediction accuracy, while also improving model interpretability and practical applicability [26].

Recently, Transformer architectures and their variants have demonstrated advantages in RUL prediction. Z. Fan et al. [27] proposed a hierarchical Transformer framework based on two-stage attention (STAR), which obtained valuable information at different time scales by capturing temporal attention and sensor variable attention respectively. Moreover, recent studies have begun to explore multi-feature fusion strategies, and the effective integration of spatial features, temporal features and statistical features has become a key factor to improve the prediction accuracy [28].

Beyond purely data-driven architectures, recent studies have also explored deep learning frameworks that incorporate prior knowledge or domain constraints into model design. Knowledge-guided learning strategies and physics-informed modeling approaches integrate degradation mechanisms or engineering knowledge into neural networks [29–32], thereby improving model reliability and generalization capability in complex industrial environments. Furthermore, recent research has begun exploring strategies such as multimodal fusion, and digital twins [33,34] and hybrid modeling approaches that combine data-driven deep learning with physical models or domain knowledge. By integrating prior physical knowledge or virtual-physical mapping mechanisms, these approaches significantly improve the reliability and engineering credibility of fault diagnosis and RUL prediction models.

In general, RUL prediction methods based on deep learning have evolved from simple to complex architectures, from single-model designs to multi-feature fusion, and from pure data-driven approaches to physical knowledge modeling, thereby significantly improving prediction accuracy. However, most existing studies are based on the assumption that the sensor data are complete and of high quality. When faced with real-world situations such as missing data, signal discontinuity or noise pollution caused by partial sensor failures in the actual industrial environment, the performance of these models degrades significantly. Most existing studies do not systematically investigate the robustness of the model under incomplete data conditions when verifying its performance on benchmark datasets such as C-MAPSS. Specifically, existing models such as CNN, LSTM and their hybrid variants inherently rely on complete time series to capture device degradation trajectories. Once the input sequence is intermittently missing or continuous segments are missing, their ability to model temporal dependencies is significantly impaired. In addition, although some recent studies have begun to focus on noise and uncertainty issues, end-to-end solutions designed specifically for the specific scenario of partial sensor failures remain largely unexplored. This limitation is particularly prominent in the real aero-engine operation and maintenance environment, which highlights the necessity of this study: The proposed robust RUL prediction architecture aims to fundamentally solve the performance degradation problem of data-driven models under incomplete data conditions. By integrating missing data reconstruction and RUL prediction in a unified framework, it ensures that the model maintains high prediction accuracy under partial sensor failure conditions.

### Methods for dealing with missing data in existing RUL prediction studies

In real-world industrial environment, sensors may be partially damaged or signal lost due to complex flight environment, severe mechanical vibration and thermal stress, resulting in incomplete or inaccurate monitoring data. To address missing or noisy data, existing studies generally adopt two main categories of solutions, namely data imputation and model robustness enhancement.

### Data imputation

Data imputation is the most direct and widely used strategy to deal with missing data, which aims to recover complete sensor data sequence through the algorithm to provide a complete input for subsequent prediction. Traditional approaches include direct deletion and statistical imputation methods. The direct deletion method can be applied when the missing ratio is small. However in RUL prediction tasks, direct deletion easily leads to sample selection bias and destroys data continuity, resulting in misleading interpretation of patterns and trends [35]. Statistical imputation methods, such as mean imputation, regression imputation, and random forest imputation, attempt to infer missing values using statistical information such as data distribution patterns and correlations. For example, Li et al. [36] applied the random forest method to reconstruct missing parts in the signals such as load, impact force and amplitude and perform RUL prediction. However, due to the complex nonlinear relationship among multi-sensor variables, traditional statistical imputation methods are often difficult to fully capture these correlations, which may limit the accuracy and reliability of reconstruction results.

In recent years, with the development of deep learning, data reconstruction methods based on neural networks have shown significant advantages. One important class of methods is self-supervised learning, such as masked autoencoders, which randomly mask portions of the input data and train the model to reconstruct the masked part, so that the model learns to infer missing values from contextual information. Another class of methods involves generative models, such as Generative adversarial networks (GAN) [37], which learn the distribution of complete data through adversarial training between a generator and a discriminator, thereby generating realistic imputation values. The missing parameter generator proposed in this study is developed based on GAN to reconstruct missing sensor data while preserving temporal dependencies and inter-sensor correlations, thereby restoring the integrity of the feature input.

Although data imputation methods provide various technical solutions for handling missing data, they suffer from a fundamental limitation: data reconstruction and RUL prediction are typically treated as two separate stages. Traditional statistical interpolation methods often fail to capture complex spatio-temporal dependencies in multi-sensor turbofan engine data. Even the most advanced generative models, such as GANs or Transformer-based imputation models [38], lack task-oriented optimization for downstream RUL prediction tasks when used independently, which may lead to reconstructed data that does not preserve the most informative features for RUL prediction.

In addition, although self-supervised methods can learn the intrinsic data distribution, their reconstruction quality depends heavily on the scale and representativeness of the training data, which challenges their generalization ability under the complex and variable operating conditions. To address these limitations, the proposed generative regression model integrates the missing-parameter generator (data reconstruction) with the RUL predictor in a unified, end-to-end trainable framework. This design enables the data reconstruction process to directly receive feedback from the RUL prediction objective, thereby ensuring that the reconstructed sensor data is not only structurally consistent, but also rich in degradation information that is crucial for accurate estimation of the remaining useful life.

### Enhanced model robustness

In addition to data imputation methods, another important class of approaches aims to enhance the robustness of the predictive model itself to missing data. Instead of relying on explicit pre-data reconstruction steps, such methods attempt to enable models to learn directly from incomplete data with missing values and maintain stable predictive performance through improved model architectures, training strategies, or loss functions. The core idea is to treat missing data as an inherent property of the data or a form of noise within the data, and design machine learning models that are robust to such conditions.

Adaptive model architecture is one of the mainstream directions to improve robustness. These methods designs models that can dynamically adapt to different missing patterns. Among them, the “fully adaptive” regressor trains an independent prediction model for each possible combination of missing features to achieve optimal performance [39]. However, when the feature dimension is high, the possible combinations of missing patterns grow exponentially, making this approach computationally infeasible. Another approach is to design the prediction that can inherently adapt to different missing patterns. For example, Le Morvan et al. [40] propose a customized neural network architecture to efficiently approximate such a fully adaptive regressor. Masking mechanism and representation learning constitute another effective path. By introducing a masking mechanism inside the model and explicitly providing the missing pattern (i.e., which features are missing) as information to the model, the model can be guided to learn a more robust data representation. For example, masked autoencoders (MAE) learn to infer missing values from contextual information by randomly masking a part of the input data and training the model to reconstruct the masked part [41]. This idea is further extended in a self-supervised mask spatial distribution learning method proposed in 2025 [29], designed for mechanical RUL prediction, which learns the spatial distribution characteristics of sensor data through a mask and reconstruction process. It has been shown that the design of masking strategies (e.g., proportional masks [41], timing-specific masks [42]) has a decisive impact on the quality and robustness of the representations ultimately learned by the model. Regularization and optimization strategies are also widely used to improve the robustness of the model. The decision boundary learned by the model can be forced to be robust to missing data perturbations by introducing appropriate regularization constraints in the design of the loss function or in the training process. For example, some studies have proposed to use a Masked denoising Autoencoder with L2 norm regularization (Masked DAE) to deal with missing data in software effort estimation, and the results show that this method can reduce the model variance, resulting in improved generalization performance [43]. In addition, from an optimization perspective, some studies treat missing data prediction as a two-stage adaptive optimization problem and propose an adaptive linear regression model, in which the regression coefficients are adaptively adjusted according to the set of observed features [39].

Although the above robustness enhancement methods improve the tolerance of incomplete data from different perspectives, they share some fundamental limitations. Firstly, such methods tend to be a “compensatory strategy” in nature. Instead of proactively addressing the underlying missing information problem, they mainly focus on encouraging the model to ‘adapt” or “ignore” missing data. Second, this compensatory approach limits the upper bound of model robustness. The performance of these methods can still deteriorate dramatically when the percentage of missing data is too high and the missing pattern is too complex (e.g., when multiple sensors fail continuously in a turbofan engine for a long period of time). Finally, the performance of many methods based on mask training is highly dependent on how well the mask policy matches the true missing pattern. If the training strategy of random masking fails to effectively simulate the complex missing patterns in the real world, the robustness of the model will be greatly reduced when deployed in real-world scenarios. These limitations highlight the motivation and novelty of this study: the proposed novel robust generative-regression model achieves a paradigm shift from “compensatory strategies” to “proactive repair” by placing generative data reconstruction and RUL prediction in an end-to-end optimization framework. Our model not only improves the robustness to missing data, but also actively reconstructs sensor data consistent with physical constraints and degradation patterns, thereby bridging the information gap, thus achieving significantly better prediction accuracy and reliability under severe conditions of partial sensor failures.

## Methods

### Dataset

#### Dataset description.

The Commercial Modular Aerospace Propulsion System Simulation (C-MAPSS) dataset released by the National Aeronautics and Space Administration (NASA) is adopted as a benchmark in this study. The dataset is generated by a high-fidelity model of a turbofan engine, which simulates the gradual degradation of the engine from a healthy state to final failure under different flight conditions (altitude, Mach number). It is a widely recognized benchmark in the field of aero-engine prognostics and health management (PHM), and is extensively used for the development and benchmarking of aero-engine RUL prediction algorithms.

The C-MAPSS dataset contains four different subsets (FD001, FD002, FD003, FD004) with varying complexity. To clearly demonstrate the core principles of the method and ensure the comparability with existing studies, this study mainly focuses on the FD001 subset, and the method can be similarly extended to other more complex subsets. The FD001 subset simulates the engine degradation process under single operating condition and single fault condition. The subset consists of a training set and a test set. The training set contained complete multivariate time series data of 100 engine units from initial healthy state to failure. The test set, in turn, contains the truncated operation sequences of another 100 engine units prior to an unknown failure time. The true remaining useful life (RUL) value of each engine unit corresponding to the test set is provided separately as the baseline truth value, which is used to evaluate the accuracy of the prediction model.

From the data structure, one record is collected for each operating cycle, that is, one time step, of each engine unit. Each data contains 24 monitoring variables, which can be divided into two categories: the first category is the three operating conditions setting parameters, representing the flight conditions, such as flight altitude, Mach number, and throttle parser Angle. The second category consists of 21 sensor measurements that represent the performance status of the engine, such as temperature, pressure, speed and flow at different locations (See Table 1 for details). These sensor readings are the core features of our model for health status assessment and RUL prediction.

**Table 1:** List of monitored sensor parameters serving as multivariate time-series inputs for the proposed model.

NO.	Name	Description	Unit
1	T2	Total fan inlet temperature	°R
2	T24	Total outlet temperature of low pressure compressor	°R
3	T30	Total outlet temperature of high pressure compressor	°R
4	T50	Total outlet temperature of low pressure turbine	°R
5	P2	Fan inlet pressure	psia
6	P15	Total pressure of bypass pipe	psia
7	P30	Total outlet pressure of high pressure compressor	psia
8	Nf	Fan physical speed	rpm
9	Nc	Core machine physical speed	rpm
10	Epr	Engine pressure ratio	–
11	Ps30	Outlet static pressure of high pressure compressor	psia
12	Phi	The ratio of fuel flow to the static pressure at the outlet of the high pressure compressor	pps/psi
13	NRf	Fan conversion speed	rpm
14	Nrc	Core machine conversion speed	rpm
15	BPR	Bypass channel ratio	–
16	farB	Burner oil/gas ratio	–
17	htBleed	Enthalpy of steam extraction	–
18	NF-dmd	Demand fan speed	rpm
19	PCNR-dmd	Demand fan conversion speed	rpm
20	W31	High pressure turbine cooling air flow	lbm/s
21	W32	Low pressure turbine cooling air flow	lbm/s

RUL is not directly provided in the dataset. For the training set, we calculate the RUL value corresponding to each time step based on the premise that “the RUL of the last operating cycle of each engine is 0”. In general, a piecewise linear RUL label is used, where a maximum RUL threshold is defined (e.g., 125 cycles), and the RUL remains constant until this threshold is reached, after which the RUL begins to decrease linearly to 0. This method is more in line with engineering practice.

### Dataset preprocessing

**Fig 2:**
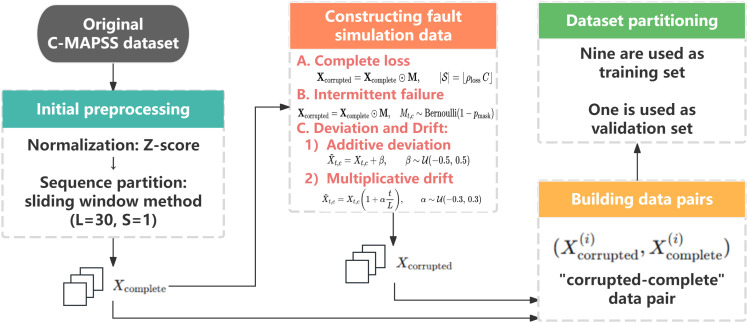
Data preprocessing workflow including sensor fault simulation and dataset construction, including initial preprocessing, simulated sensor failure generation, ten-fold dataset partitioning, and the construction of corrupted–complete sequence pairs for model training.

To facilitate the subsequent training of the proposed missing parameter generator, a dataset containing “impaired-complete” data pairs must be constructed. Since all sensor data in the original C-MAPSS dataset are complete, we design a systematic data corruption scheme to simulate real sensor failures (See Fig 2).

### Normalization and sequence partitioning

The first step of data preprocessing is to standardize the raw data of all sensor channels. Raw sensor data usually have different dimensions and numerical ranges. If they are directly input into the model, the sensor with large numerical values may dominate the training process, resulting in the model being insensitive to smaller but physically important sensor signals. To solve this problem, we normalized the readings of each sensor channel by Z-score, transforming them into a distribution with mean 0 and standard deviation 1. After this transformation, all sensor channels are unified to the same scale, which not only accelerates the convergence process of model training, but also improves the final performance of the model.

We then transform the entire life cycle data of each engine into time series samples that can be processed by the deep learning model. The operating data of the engine is a multivariate time series of variable length. We use the sliding window method to divide it into consecutive subsequences of fixed length. Given the complete sequence of an engine, we generate multiple consecutive samples by sliding with a fixed window length (*L* = 30) and step size (*S* = 1). This step transforms the time series data of variable length into structured tensors, which are ready for subsequent model input.

### Partial sensor fault simulation

Based on the normalized and sequence partitioned data, we systematically implement a series of artificial data corruption strategies to simulate a variety of partial sensor failures that may occur in the actual operating environment. These strategies aim to generate time series with different missing and bias patterns, thus constructing a collection of “corrupted-complete” data pairs for training and evaluating our robustness model.

a) Completely lost

In order to simulate hard faults with complete sensor failure or communication interruption, we employ a complete loss strategy. Specifically, for a given multivariate time series sample $X\in {\mathbb{R}}^{L\times C}\;$, where *L* is the sequence length and *C* is the total number of sensor channels, we randomly select a proportion $\rho_{loss}\;$ (e.g., 10%, 20%, 30%) of sensor channels. For each selected channel *c*, we force all its readings over the entire sequence length *L* to zero. When higher values of $\rho_{loss}\;$ is selected, this strategy simultaneously affects multiple sensor channels, thereby naturally inducing continuous missing-data scenarios with cross-sensor joint failures. This operation generates an extreme but common failure scenario where the signal from a particular sensor is completely unavailable for a certain period of time to challenge the model’s reasoning ability under persistent missing critical information.

b) Intermittent failure

However, sensor failures in practice are often not so absolute, and more common are intermittent failures caused by instantaneous interference or poor contact. To simulate such cases, we introduce a random masking strategy. The strategy operates at a more fine-grained level of time steps. For each sensor reading in the sequence, we set it to zero with an independent probability *p*_*mask*_ (e.g., 0.1, 0.2, 0.3). This process can be formalized by a randomly generated mask matrix $M\in \{0,1{\}}^{L\times C}\;$, where each element of the matrix is assigned a probability *p*_*mask*_ of 0 and a 1 otherwise. Subsequently, the damaged sequence is calculated via element-wise multiplication. Since the masking operation is applied jointly along the temporal and sensor dimensions, this strategy, in a multi-sensor setting, allows multiple sensors to fail simultaneously within local or overlapping time windows, thereby covering intermittent scenarios of cross-sensor joint failures. This approach introduces random and sparse missing points into the input data, effectively forcing the model to learn to rely on contextual information rather than readings at a single time point for estimation and prediction.

c) Deviation and drift

In addition to complete missing data, systematic deviation of readings from sensors due to calibration drift, aging, or environmental factors is another critical class of soft faults, which is representative of non-random, structured noise. To simulate such phenomena, we design a bias and drift strategy, which consists of two main modes. One is additive bias, where we randomly select the sensor channel and superimpose a fixed amount of bias β  to its readings at all time steps. This bias, β , is sampled from a preset uniform distribution, such as β~U(−0.5,+0.5) , and represents the shift from the normalized data. The second is a more subtle multiplicative drift, which is used to model the slow change of sensor sensitivity over time. For a randomly selected sensor channel, we make its readings drift linearly in time by multiplying by a factor that varies linearly in time: 1+α·t/L , where α  is the coefficient that controls the slope of the drift, sampled from another uniform distribution, e.g., α~U(−0.3,+0.3) , and *t* is the timestep index. The additive bias models a fixed measurement error, while the multiplicative drift models a progressive degradation of the sensor performance.

After the above steps, we generate the corresponding compromised version *X*_*corrupted*_ for each complete engine run sequence *X*_*complete*_. Finally, we build the training dataset as a set of pairs:


$$D_{train}={\left\{\left(X_{corrupted}^{\left(i\right)},X_{complete}^{\left(i\right)}\right)\right\}}_{i=1}^{N}\;$$ (1)

Where $X_{corrupted}^{\left(i\right)}\;$ is the input and $X_{complete}^{\left(i\right)}\;$ is the target that the missing-parameter generator is required to to reconstruct. During training, we mix all the above failure modes and randomly vary their severity parameters (such as loss ratio, mask probability, bias size), so that the model can adapt to a variety of complex and unforeseen sensor failure situations. This diverse exposure training is critical, driving the model to learn not only to repair specific missing patterns, but also to learn the underlying temporal dynamics and intercorrelations of sensor data, thus gaining strong generalization ability to deal with unseen failure situations. For model evaluation, a set of fixed failure modes and parameters are used to ensure the fairness and consistency of performance comparison.

### Missing-parameter generator

To achieve high-quality data repair under partial sensor failure conditions, we build a missing parameter generator, which is trained on the Wasserstein Generative Adversarial Network (WGAN) framework. Gradient Penalty (GP) strategy is adopted to enforce Lipschitz constraints, and the combined model is often referred to as WGAN-GP. Known for its excellent training stability, this framework can effectively learn complex high-dimensional data distributions, which is well suited for our time-series data repair task.

The WGAN-GP framework consists of a generator G and a discriminator D (often referred to as a Critic in WGAN). The core idea is to measure the difference between the generated data distribution PG and the true data distribution *P*_*data*_ by optimizing the Wasserstein distance. Compared to the loss function of the original GAN, the Wasserstein distance provides smoother and more informative gradients, which significantly alleviates mode collapse and training instability. The optimization objective of WGAN can be formulated as follows:


$$\min_{G}\max_{D\in 𝒟}{𝔼}_{x\sim P_{data}}\left[D(x)\right]-{𝔼}_{\tilde{z}\sim P_{G}}\left[D\left(\tilde{z}\right)\right]\;$$ (2)

where 𝒟  is the set of 1-Lipschitz functions. To ensure that the discriminator *D* satisfies the Lipschitz constraint, GP strategy proposed to add a gradient penalty term to the loss function instead of weight clipping used in the original WGAN. Therefore, the objective function of the discriminator is extended as follows:


$$L_{D}={𝔼}_{\tilde{z}\sim P_{G}}\left[D\left(\tilde{z}\right)\right]-{𝔼}_{x\sim P_{data}}\left[D(x)\right]+\lambda \cdot {𝔼}_{\hat{x}\sim P_{\hat{x}}}\left[{\left({\left\|\nabla_{\hat{x}}D\left(\hat{x}\right)\right\|}_{2}-1\right)}^{2}\right]\;$$ (3)

In this formula, $\hat{x}\;$ is a random interpolation point on the line between the real data sample *x* and the generated sample $\tilde{z}\;$, that is, $\hat{x}=\epsilon x+(1-\epsilon )\tilde{z}\;$, where ϵ~U[0,1] . The hyperparameter λ  is used to control the weight of the gradient penalty term.

Although WGAN-GP provides a stable training framework, we make critical adaptations to its network architecture and loss function to serve the specific task of time series data repair for RUL prediction. We replace with convolutional architectures commonly used for image generation and design architectures for the generator *G* and discriminator *D* specifically to handle multivariate time series.

The generator *G* receives the compromised multivariate time series $X_{corrupted}\in {\mathbb{R}}^{L\times C}\;$ as input. The network architecture adopts an encoder-decoder architecture, and the encoder part is composed of multi-layer bidirectional Long Short-Term memory (Bi-LSTM) network, which aims to capture the implicit inter-sensor correlations and high-level temporal patterns from the context of the damaged sequence. The decoder is composed of several fully connected layers, which are responsible for mapping the encoded feature vector back to the original sequence space, and the output is the complete data reconstruction result *X*_*reconstructed*_ with dimension *L* × *C*.

Discriminator *D* is designed as a time series discriminator based on one-dimensional convolutional neural Network (1D-CNN) combined with LSTM. It takes a time series (either the real complete sequence *X*_*complete*_ or the missing-parameter generator repaired sequence *X*_*reconstructed*_) and passes it through stacked convolutional layers to capture local dependency patterns, through LSTM layers to understand long-term dynamics, and finally through a linear layer to output a scalar score. This score is not a true/false probability in the traditional sense, but rather a measure of “truth” that the input sequence belongs to the true data distribution, i.e., the critic value in the Wasserstein distance.

On the overall loss function, simply optimizing the Wasserstein distance (i.e., adversarial loss) may generate data that is statistically reasonable but deviates from the true degradation trajectory. To ensure that the repaired data has high fidelity and utility for the downstream RUL prediction task, we introduce an explicit supervision signal in the loss function of the missing-parameter generator:


$$L_{G}=-{𝔼}_{X_{corrupted}\sim P_{G}}\left[D\left(G\left(X_{corrupted}\right)\right)\right]+\eta \cdot {\left\|M⊙\left(G\left(X_{corrupted}\right)-X_{complete}\right)\right\|}_{2}^{2}\;$$ (4)

Here, we introduce a mask matrix *M* whose elements set to one ones where data is missing or corrupted and zeros elsewhere. The regression loss term guides the missing-parameter generator to focus on accurately repairing missing or damaged parts of the data, rather than modifying correct readings. The hyperparameter η  is used to balance the distributional learning ability of the adversarial objective with the numerical accuracy of the regression objective.

With this design, the discriminator *D* not only evaluates the “truthfulness” of the generated sequence, but also indirectly evaluates its “potential utility” for the prediction task. The generator *G*, in turn, is guided to retain and recover the discriminative features most relevant to the device degradation process while learning to generate realistic data. This collaborative optimization process ultimately results in a smart missing parameter generator optimized specifically for the robustness of RUL predictions.

## RUL predictor

The complete sequence *X*_*reconstructed*_ repaired by the missing parameter generator is input into a RUL predictor based on a hybrid architecture of convolutional neural network and Long Short-Term Memory Network (CNN-LSTM) to achieve the end-to-end estimation of the remaining useful life. This hybrid model aims to jointly utilize the powerful ability of CNN in extracting local time series patterns and the inherent advantages of LSTM in capturing long-term time dependence. The CNN-LSTM hybrid model has been widely proved to be effective in dealing with time series prediction problems. In this study, we build on this and perform specific optimization for the engine degradation prediction task.

The core idea of the CNN-LSTM hybrid architecture is to divide the feature extraction process into two levels. First, the CNN acts as a powerful local feature extractor, acting on the input time series data. Specifically, we treat the repaired multivariate time series $X_{reconstructed}\in {\mathbb{R}}^{L\times C}\;$ as a one-dimensional input of length *L* and the number of channels *C*. The model then processes it using multiple 1D-Convolutional Layers. Each convolutional layer slides in the time dimension through its convolution kernel, so as to capture the early signs of degradation such as local fluctuations, trends and abrupt changes in sensor readings within a short time window. The feature map after convolution operation introduces nonlinearity through the Rectified Linear Unit (ReLU) activation function, and can be downsampled by a one-dimensional maxpooling layer (1D-MaxPooling) to enhance the robustness of the feature and reduce the computational complexity. After stacked convolution and pooling layers, the input sequence is transformed into a high-level feature representation rich in local temporal information.

However, the degradation of an engine is a continuous process with long-term memory, and its final failure state depends on the whole operation history and not only on the recent state. To model this long-term dependency, we feed the high-level feature sequence output by the CNN into the LSTM network. LSTM can effectively learn long-term dependencies due to its unique gating mechanism (input gate, forget gate, output gate), which avoids the gradient vanishing or explosion problem that may occur in traditional recurrent neural networks during training. In the proposed architecture, the LSTM layer receives the feature sequence processed by the CNN, and based on its internal state cells, integrates context information over the entire sequence length, so as to understand the overall trajectory of degradation and the dynamic evolution law.

Eventually, the LSTM hidden state of the last time step is extracted, which is thought to encode the condensed degradation information of the whole sequence. This hidden state is then fed to a fully connected regression layer that outputs a single scalar value, the estimated remaining useful life ${\hat{y}}_{RUL}\;$ of the engine unit corresponding to this input sequence.

The training objective of the RUL predictor is to minimize the difference between its predicted value and the true RUL value. We adopt the mean squared error (MSE) as the loss function, which is a standard choice in regression tasks. Given a batch containing *N* samples, the loss function *L*_*RUL*_ is defined as follows:


$$L_{RUL}=\frac{1}{N}\sum_{i=1}^{N}{\left(y_{RUL}^{\left(i\right)}-{\hat{y}}_{RUL}^{\left(i\right)}\right)}^{2}\;$$ (5)

where $y_{RUL}^{\left(i\right)}\;$ is the true RUL value of the *i* sample and ${\hat{y}}_{RUL}^{\left(i\right)}\;$ is the predicted value of the model.

### Model training and optimization

#### Overall loss function and training objective.

The proposed framework contains two trainable modules: a missing parameter generator *G* and a RUL predictor *P*. Its training objective is achieved by a hybrid loss function that combines an adversarial loss, a data reconstruction loss, and a regression loss. This joint loss function ensures that the missing-parameter generator can not only produce data that fits the true data distribution, but also preferentially retain discriminative features that are crucial for the RUL prediction task.

The total loss function *L*_*G*_ of a generator *G* is the weighted sum of its adversarial loss $L_{G}^{adv}\;$ and task-oriented regression loss $L_{G}^{rec}\;$ as follows:


$$L_{G}=L_{G}^{adv}+\eta \cdot L_{G}^{rec}\;$$ (6)

Here, $L_{G}^{adv}=-{𝔼}_{X_{corrupted}}\left[D\left(G\left(X_{corrupted}\right)\right)\right]\;$ is the standard adversarial loss of the generator in the WGAN-GP framework, which aims to maximize the score of the discriminator on the generated data. $L_{G}^{rec}=𝔼\left[{\left\|M⊙\left(G\left(X_{corrupted}\right)-X_{complete}\right)\right\|}_{2}^{2}\right]\;$ is the masked mean squared error loss, which forces the missing-parameter generator to reconstruct the data at the damaged locations exactly, and *M* is the binary mask matrix identifying the missing or damaged locations. The coefficient η  is used to balance these two objectives. Specifically, a smaller value of η  encourages the missing-parameter generator to focus more on distribution consistency through adversarial learning, whereas a larger value emphasizes the accurate reconstruction of corrupted sensor measurements. In this work, η  is treated as a learnable parameter and optimized together with the network parameters via backpropagation. This adaptive balancing mechanism allows the model to dynamically adjust the trade-off between distribution realism and reconstruction fidelity during training, thereby improving convergence stability and enhancing the robustness of the repaired signals.

The loss function *L*_*P*_ for the RUL predictor *P* is the mean squared error (MSE) between the predicted and true values:


$$L_{P}=\frac{1}{N}\sum_{i=1}^{N}{\left(y_{RUL}^{\left(i\right)}-P\left(G\left(X_{corrupted}^{\left(i\right)}\right)\right)\right)}^{2}\;$$ (7)

In the end-to-end joint fine-tuning stage, the overall optimization objective is to minimize the following joint loss *L*_*total*_:


$$L_{total}=\lambda_{1}\cdot L_{G}+\lambda_{2}\cdot L_{P}\;$$ (8)

where $\lambda_{1}\;$ and $\lambda_{2}\;$ are hyperparameters that control the relative weight of the two losses. Through this joint loss, the gradient is backpropagated from the RUL predictor to the missing-parameter generator, which guides the missing-parameter generator to perform data repair that is optimal for the final prediction task.

### Training process

The model is trained using a staged strategy to ensure stable convergence. In the first stage, we fix the generator *G* and discriminator D and train the RUL predictor *P* independently using the mean squared error loss *L*_*P*_. This stage trains the complete data part in the artificially constructed “damage-complete” data pair, aiming to provide a good initial weight for the predictor. In the second stage, we fix the predictor *P* and focus on training the generator *G* and discriminator *D*. In this stage, we use the loss functions *L*_*P*_ and *L*_*D*_ to alternately optimize the missing-parameter generator and discriminator so that the missing-parameter generator learns to generate high-quality reconstructed data. In the third stage, the end-to-end joint fine-tuning stage, we connect the generator *G* and the pre-trained predictor *P* to minimize the joint loss *L*_*total*_ with a small learning rate. In this stage, the parameters of the two modules are adjusted collaboratively, and the overall performance is finally optimized.

All training was performed using the Adam optimizer with momentum parameters set to $\beta_{1}=0.5\;$ and $\beta_{2}=0.999\;$. The learning rate was set to 1e-4, and the polynomial learning rate decay strategy was used to effectively suppress the overfitting phenomenon. The batch size was set to 256.

To further illustrate the optimization workflow, the three-stage training procedure of the proposed framework is summarized in S1 Fig.

### Ten-fold cross validation and statistical validation

To obtain a robust performance evaluation, we employ ten-fold cross validation. The training set is evenly divided into 10 subsets, one of which is alternately used as the validation set and the remaining nine are used for training, and the process is repeated 10 times to ensure that all data are used for validation.

Based on the ten-fold validation results, we further perform statistical significance tests. The paired samples t-test was used to compare the prediction errors between the model of this study and each baseline method, and their p-values were calculated. A difference in performance is considered statistically significant when the p-value is less than the significance level (α=0.05 ). At the same time, 95% confidence intervals for the improvement of the performance metrics are reported to quantify the range of uncertainty of the difference. This analysis provides statistical evidence for the significant superiority of model performance.

### Experiments

#### Experimental environment.

To ensure the fairness and reproducibility of the experimental results, all experiments were conducted under a standardized hardware and software environment. The hardware setup included a server equipped with an Intel^®^ Xeon^®^ Platinum 8352V CPU (2.10GHz) and an NVIDIA RTX 4090 GPU with 24GB of memory. On the software side, the environment was built on Ubuntu 18.04, using Python 3.8 and PyTorch 2.4 as the core programming language and framework.

#### Evaluation indicators.

To evaluate the comprehensive performance of the proposed framework, this study established a systematic evaluation system from three key dimensions: the quality of generated data, the accuracy of RUL prediction, and the computational efficiency of the model. The results for all metrics will be presented based on ten-fold cross validation and their “mean±standard deviation” will be reported. We will subsequently compare the results of the proposed model with the baseline method using a paired samples t-test and report p-values with 95% confidence intervals to verify the statistical significance of the performance improvement.

#### Generate quality evaluation indicators.

The quality of data repair directly affects the reliability of subsequent RUL prediction. To accurately quantify the quality of reconstructed data from the missing parameter generator, we adopt the following two complementary evaluation indicators, which are evaluated from two perspectives of numerical accuracy and structural similarity, respectively.

**Root Mean Square Error (RMSE).** This index is the core index to measure the numerical deviation between the reconstructed data and the true complete data. The calculation focuses on all data points that have been corrupted by a simulated fault and is defined as follows:


$$RMSE_{gen}=\sqrt{\frac{1}{N_{m}}\sum_{i=1}^{N_{m}}{\left(y_{i}-{\hat{y}}_{i}\right)}^{2}}\;$$ (9)

where *y*_*i*_ represents the true sensor reading, ${\hat{y}}_{i}\;$ represents the reconstructed value of the missing-parameter generator, and *N*_*m*_ is the total number of all missing or damaged data points. The lower RMSE value in this study means that the repaired sensor readings are closer to the real physical values, which lays a reliable data foundation for accurate health status assessment.

**Structural Similarity Index Measure (SSIM).** Although RMSE can effectively reflect numerical errors, it has limited perception of signal waveform structure. As an index more consistent with human visual perception, SSIM is able to evaluate the similarity of two segments of signals in terms of structural information, brightness and contrast. For each repaired sensor-channel time series *x* and its corresponding true complete sequence *y*, its computation is defined as follows:


$$SSIM(x,y)=\frac{\left(2\mu_{x}\mu_{y}+C_{1}\right)\left(2\sigma_{xy}+C_{2}\right)}{\left(\mu_{x}^{2}+\mu_{y}^{2}+C_{1}\right)\left(\sigma_{x}^{2}+\sigma_{y}^{2}+C_{2}\right)}\;$$ (10)

Here, $\mu_{x}\;$ and $\mu_{y}\;$ are the means of the sequences *x* and *y*, respectively, $\sigma_{x}^{2}\;$ and $\sigma_{y}^{2}\;$ are their variances, and $\sigma_{xy}\;$ is their covariance. The constants *C*_1_ and *C*_2_ are used to avoid the denominator being zero and maintain computational stability. The SSIM ranges from [−1, 1], and the closer its value is to 1, the better the missing-parameter generator reconstructs key fluctuations, trends, and abrupt patterns in sensor readings, which are often important structural information for identifying early fault symptoms.

### Regression prediction evaluation indicators

The accuracy of RUL prediction is directly related to the effectiveness of predictive maintenance decisions, and we adopt the following standard metrics that are widely recognized in the PHM field.

**Root Mean Square Error (RMSE).** This is the core metric for assessing the absolute accuracy of RUL predictions. It is calculated based on the final RUL prediction results of all tested engine units:


$$RMSE_{RUL}=\sqrt{\frac{1}{N}\sum_{i=1}^{N}{\left(RUL_{true}^{\left(i\right)}-RUL_{pred}^{\left(i\right)}\right)}^{2}}\;$$ (11)

Here, *N* is the number of tested engines, $RUL_{true}^{\left(i\right)}\;$ and $RUL_{pred}^{\left(i\right)}\;$ are the true and predicted RUL of the *i* engine, respectively. This index is more sensitive to large prediction errors and can effectively reveal the prediction stability of the model.

**Score function (Score).** In order to conform to industry practice and facilitate a fair comparison with other similar studies, we also use the scoring function provided by NASA:


$$Score=\sum_{i=1}^{N}S_{i},\;S_{i}=\left\{ \begin{array}{cc} \exp\left(-\frac{d_{i}}{10}\right)-1, & d_{i}\leq 0\\ \exp\left(\frac{d_{i}}{13}\right)-1, & d_{i}>0 \end{array}\right.\;$$ (12)

Here, $d_{i}=RUL_{pred}^{\left(i\right)}-RUL_{true}^{\left(i\right)}\;$. A lower Score indicates better and safer overall prediction performance.

### Model calculation cost index

In practical aero-engine monitoring applications, the real-time performance and deployment feasibility of the model are equally important, so we measure the following key computational efficiency and complexity indicators at the same time:

**Number of Parameters of the Model.** Report the total number of trainable parameters for the missing-parameter generator and predictor, in millions. This metric directly reflects the complexity and memory footprint of the model. The model with smaller parameters is more suitable for deploying on edge devices to achieve real-time inference.

**Average Training Time.** The average total time required to complete all training phases of the model (including pre-training and joint fine-tuning) under a specific hardware configuration is recorded. This metric reflects the efficiency of model development. Although training is usually done in the cloud, reasonable training time helps to quickly iterate and optimize the model architecture, speeding up the technology development cycle.

**Average Inference Time per Sample.** Records the average time it takes for the model to complete the whole process from data repair to RUL prediction for a single test sequence. This index is the key to evaluate whether the model can meet the needs of online real-time monitoring and forecasting.

### Performance analysis of the missing parameter generator

To deeply evaluate the performance of the missing parameter generator, we performed a systematic analysis of its data repair capability under different sensor failure rates, using RMSE and SSIM to assess imputation quality from the complementary perspectives of numerical accuracy and temporal-structure consistency respectively. As shown in Table 2, the missing parameter generator exhibits excellent data repair capability under different levels of sensor failure conditions. It is worth noting that even at a higher failure rate of 30%, the reconstructed RMSE can still be controlled within 0.19, and the SSIM remains above 0.90, which indicates that the repaired data are highly consistent with the real data in terms of time series morphology and dynamic characteristics. This ability to maintain the integrity of the data structure is essential to accurately capture critical patterns during engine degradation.

**Table 2:** Imputation performance of the missing parameter generator under different failure rates.

Sensor failure rate	RMSE	SSIM
10%	0.08±0.02	0.97±0.01
20%	0.13±0.02	0.95±0.02
30%	0.19±0.03	0.91±0.02

In-depth analysis shows that the performance degradation of the missing-parameter generator has a sublinear relationship with the failure rate, which indicates that the generator has good fault tolerance. For example, when the failure rate increases from 10% to 30%, the reconstruction RMSE only increases by about 2.3 times instead of the theoretical 3 times relationship, reflecting the robustness of the model under extreme conditions.

To further verify the superiority of our proposed missing parameter generator, we compare it with several traditional data repair methods commonly used in industry. As shown in Table 3, taking the 20% sensor failure rate as an example, the proposed generator significantly outperforms the conventional method in all metrics. The statistical significance test results show that there are significant differences between the proposed generator and all traditional imputation methods in all key indicators (*p* < 0.01). The 95% confidence interval of the performance improvement of the proposed generator compared with the traditional best method matrix completion is [0.10, 0.15], which is completely in the positive range and far from the zero value, further confirming the reliability of the improvement. Furthermore, compared with Transformer-based imputation method which is widely adopted in recent years, the proposed approach also achieves an improvement in RMSE, with a 95% confidence interval of [0.06, 0.09] (*p* < 0.01). These results indicate that although Transformer-based imputation model can outperform traditional statistical methods, its reconstructed data remain limited in characterizing degradation information.

**Table 3:** Comparison of different data repair methods for multivariate time-series imputation under a 20% simulated sensor failure conditions based on reconstruction accuracy (RMSE, SSIM) and statistical significance.

Repair method	RMSE	SSIM	vs. Ours (p-value)	95% CI
Linear interpolation	0.32±0.04	0.83±0.04	<0.001**	[0.17, 0.21]
KNN interpolation	0.28±0.03	0.86±0.03	<0.001**	[0.14, 0.17]
Low rank matrix completion	0.25±0.03	0.88±0.03	0.003**	[0.10, 0.15]
Transformer-based imputation	0.20±0.03	0.91±0.02	0.009	[0.06, 0.09]
**Ours**	**0.13±0.02**	**0.95±0.02**	–	–

### SOTA comparative analysis of RUL prediction

To fully evaluate the advancement of the proposed framework, we compare it with the representative methods in the field of turbofan engine RUL prediction in recent years. As shown in Table 4, our proposed model shows the best performance in the two key metrics of RMSE and Score. Specifically, compared with benchmark models such as CNN, LSTM, Transformer, CAELSTM and CNN-Bi-LSTM, the P-values of all comparisons were less than 0.05, among which four comparisons reached the extremely significant level of *p* < 0.001. The 95% confidence interval analysis further confirmed this conclusion. The lower and upper limits of all intervals were greater than zero, indicating that the performance improvement has a high degree of statistical reliability. Especially when compared with the best-performing baseline model CNN-Bi-LSTM, although the gap between the two was relatively small, it still reached a statistically significant level (*p* = 0.018), and the confidence interval [0.07, 1.07] was completely within the positive range. These results collectively demonstrate that the framework proposed in this study has indeed achieved significant and reliable performance improvements in the task of predicting the remaining useful life of turbofan engines.

**Table 4:** Comparison of RUL prediction performance between the proposed framework and representative deep learning models under sensor fault conditions, evaluated using RMSE, standard C-MAPSS scoring function and statistical significance.

Models	RMSE	Score	vs. Ours (p-value)	95% CI
CNN [44]	18.45	1286.70	<0.001**	[5.92, 7.68]
LSTM [45]	14.53	322.44	<0.001**	[1.98, 2.78]
Transformer [46]	13.52	287.07	0.002**	[0.87, 1.87]
CAELSTM [6]	14.44	282.38	<0.001**	[1.89, 2.69]
CNN-Bi-LSTM [47]	13.22	232.24	0.018*	[0.07, 1.07]
**Ours**	**12.15**	**228.47**	–	–

### Interpretability analysis of generator-repaired data

To investigate the interpretability of the missing-parameter generator within the proposed framework, we analyze its repair behavior from temporal and task-aware perspectives, with a particular focus on identifying the critical repair periods that contribute most to RUL prediction. This analysis is conducted in a post-hoc manner by jointly examining the damaged input sequence $\tilde{X}\;$ and its generator-repaired counterpart *X*_*reconstructed*_. Specifically, we compute the repair magnitude along the temporal dimension by aggregating the reconstruction changes across all sensor channels at each time steps. The resulting repair-magnitude curve reflects the extent of the missing-parameter generator’s intervention over time, thereby revealing the temporal regions where repair actions are primally concentrated. Peaks in this amplitude curve indicate periods where the generator performs stronger corrections on corrupted signals, suggesting that these temporal regions contain critical degradation information that requires reconstruction.

Furthermore, we assess the task relevance of different repair periods by analyzing how repair behaviors in distinct temporal intervals affect downstream RUL prediction performance. To further illustrate the repair mechanism, we also examine representative sensor channels by qualitatively comparing the time-series waveforms before and after repair. In several degradation-sensitive sensors, corrupted segments typically appear as discontinuities or flattened signal patterns due to missing or distorted readings. After reconstruction by the missing-parameter generator, these segments are restored into smooth trajectories that are consistent with the surrounding temporal context and the overall degradation trend. This waveform-level observation helps explain how the generator reconstructs physically plausible degradation dynamics rather than merely filling missing values.

The results indicate that repairs applied during the late operational stages of engine life have a substantially greater impact on improving RUL prediction accuracy. In contrast, repair actions in earlier stage where the system remains in a relatively stable and healthy condition, contribute less to the final prediction performance. This phenomenon is consistent with the physical characteristics of degradation processes in real-world machinery, where late-stage degradation signals are more informative and directly associated with the RUL.

### Ablation experiments

Ablation experiments verify the contribution of key modules to the overall model performance. In this study, in order to verify the key role of the missing parameter generator, we compare the performance difference between the full model and the variant model with the missing parameter generator removed. In the variant model, we directly use the compromised sensor data input into the RUL predictor for training and testing without any data repair process.

The ablation experiment results in Table 5 clearly demonstrate the important value of the missing parameter generator. When this component is removed, the performance of the model deteriorates 39.0% in RMSE compared to the full model, and the Score function value also increases significantly. This result indicates that the missing parameter generator is decisive for maintaining the accuracy of the RUL prediction system under partial sensor failure conditions.

**Table 5:** Ablation study evaluating the impact of the missing-parameter generator on RUL prediction performance under sensor failure conditions.

Model configuration	RMSE	Score	Performance degradation
Variant model (generator without missing parameters)	16.89	376.12	+39.00%
**Full model**	**12.15**	**228.47**	–

The detailed analysis shows that the missing parameter generator can effectively recover the critical degradation information in the damaged sensor data through its precise data repair mechanism. The generator not only fills in the missing values, but also preserves the complex physical correlation and temporal dynamic characteristics between sensor data. In contrast, the direct use of damaged data for prediction is easy to cause the model to receive distorted and one-sided health status information, which in turn leads to the degradation of prediction performance.

Building on the ablation results, we further analyze the interaction mechanism between the imputation quality of the missing-parameter generator and the downstream RUL prediction performance, which clarifies how the quality of reconstructed sensor signals influences degradation feature learning and final RUL estimation. The analysis demonstrates a strong positive correlation exists between them, indicating that the effectiveness of the missing-parameter generator directly affects the performance of the prediction model. The ablation results not only verify the necessity of the missing-parameter generator within the overall framework but also reveal the direct impact of imputed data quality on RUL prediction accuracy. From a causal perspective, imputation errors do not remain as local numerical deviations of the multivariate time-series data, but instead propagate and accumulate along the temporal dimension, thereby interfering with the model’s ability to accurately characterize key degradation patterns such as degradation onset, degradation rate, and overall degradation trends. As a result, inaccurate reconstruction may introduce distorted health-state information, which further affects the degradation feature representation learned by the CNN–LSTM predictor and ultimately reduces prediction accuracy.

When combined with the imputation performance evaluation results, it can be observed that the missing-parameter generator is able to preserve the structural consistency and temporal dynamics of time-series data under different sensor failure rates, as reflected by relatively high SSIM values. Compared with point-wise reconstruction accuracy alone, the RUL prediction model is more sensitive to the continuity of key degradation stages and the preservation of inter-sensor correlations in the imputed data. When these critical characteristics are reliably recovered by the missing-parameter generator, the prediction performance can remain stable even under relatively high levels of data missingness.

### Analysis of computational efficiency of the model

In order to evaluate the deployment feasibility of the proposed framework in a real industrial environment, this subsection provides a comprehensive quantitative analysis of the computational efficiency of the model. Table 6 presents the detailed metrics of the proposed full model in terms of computational efficiency.

**Table 6:** Computational efficiency and deployment-related complexity of the proposed model, including model size, training time, and inference latency measured on an NVIDIA RTX 4090 GPU.

Evaluation metrics	Value	Notes
Model parameters	7.8 M	The missing-parameter generator is 4.9M and the predictor is 2.9M
Average training time	5.5 hours	NVIDIA RTX 4090 with a complete training cycles
Average single-sample inference time	18.3 ms	It includes the whole process of data repair and RUL prediction

In terms of model complexity, the complete framework contains a total of 7.8M trainable parameters, of which the missing parameter generator accounts for 4.9M parameters and the RUL predictor accounts for 2.9M parameters. This parameter magnitude reflects the good balance we have achieved between model performance and complexity. The distribution of the number of parameters also reflects the relative importance of the two components, with the missing-parameter generator requiring more parameters to model complex sensor data distributions and timing dependencies.

Training efficiency analysis shows that the full model takes about 5.5 hours to converge on NVIDIA RTX 4090 hardware environment. This training time takes into account all training phases including adversarial training of WGAN-GP, supervised learning of the predictor, and end-to-end joint fine-tuning. Despite the relatively long training time, this mainly stems from the inherent stability requirements of generative adversarial network training and the complexity of the joint optimization process. In practical engineering applications, model training is usually carried out offline, and this time cost is completely acceptable.

Inference efficiency is a key factor in determining whether the model can be deployed in real industrial scenarios. The test results show that the model only takes 18.3 milliseconds on average to complete the whole process from data repair to RUL prediction for a single test sample. This excellent inference speed is mainly due to the optimized design of the model architecture, including the efficient local feature extraction of the CNN layer and the sequence processing ability of the LSTM layer. Considering that the typical data sampling interval of the aero-engine health monitoring system is usually on the order of 1 second, the inference speed of the proposed model has more than 50 times the real-time margin, which provides sufficient technical feasibility for the deployment on resource-constrained edge computing devices.

Comprehensive computational efficiency analysis shows that the proposed robust generative regression framework not only achieves the advanced level in prediction performance, but also fully meets the requirements of industrial deployment in terms of computational efficiency. Moderate model parameters ensure the deployability on common hardware platforms, and excellent inference speed supports real-time monitoring requirements. These characteristics together form an important foundation for the framework from theoretical research to engineering applications.

## Conclusion

In this paper, we propose a novel robust Generative regression framework based on Long Short-Term Memory Generative Adversarial Network for the remaining useful life prediction of turbofan engines under partial sensor failures. Compared with the current mainstream methods in the RUL prediction field, this study achieves important methodological innovations that break through the strong dependency assumption of traditional methods on complete sensor data. By tightly integrating data repair for prediction tasks with RUL prediction in an end-to-end joint learning framework, this study provides a new technical path to solve the challenge of incomplete sensor data in practical engineering.

The core innovation of this study is to propose the concept of “data repair for prediction task”, which is embodied in the design of the missing parameter generator. The generator not only considers the complex coupling relationship and time dependence between sensors, but also introduces a task-oriented regression loss to ensure that the repaired data can retain the discriminative features related to the equipment degradation process to the maximum extent. Compared with the current repair methods based on deterministic models, our generative method can better capture the uncertainty of sensor data and produce repair results that are more consistent with the real data distribution. Ablation experiments further validate the critical role of the missing parameter generator, with removal of this component leading to a nearly 40% decrease in prediction performance, highlighting the need for intelligent data repair under sensor failure conditions.

In terms of prediction performance, the complete framework proposed in this study shows significant advantages. Compared with the existing time series models, our method achieves the state-of-the-art performance on the NASA C-MAPSS dataset, and the reported data are all statistically significant. The significant improvement of RMSE and Score function indicators indicates that our model can reduce the potential risk caused by delayed warning in actual operation and maintenance, which is of great significance for the health management of safety-critical systems such as aeroengines.

From the perspective of engineering application, this study promotes RUL prediction technology from “laboratory environment” to “industrial solution”. By effectively handling the actual situation of partial sensor failures, our framework provides technical support for building a truly robust predictive maintenance system. The inference efficiency of the model supports real-time deployment requirements, and the processing time of a single sample is only 18.3 milliseconds, which makes it have broad application prospects in industrial Internet of Things and edge computing scenarios.

With respect to generalizability, although the experimental validation is only conducted on the NASA C-MAPSS dataset, the proposed method is not designed to be restricted to a specific data distribution or operating condition. First, the proposed framework does not depend on sensor configurations, physical variable definitions, or predefined degradation function forms that are unique to the C-MAPSS dataset. Second, unlike RUL prediction models developed under idealized assumptions, the proposed approach explicitly accounts for sensor missingness and anomalies, which are prevalent in real-world industrial scenarios, thereby enhancing robustness under incomplete observations. Furthermore, from a methodological standpoint, the proposed generative-predictive integration paradigm is largely task-agnostic. For other types of equipment and industrial systems, as long as their operational states can be represented as multivariate time series and the RUL or health state exhibits a learnable degradation trend over time, the framework retains broad applicability through retraining or lightweight fine-tuning. Future work will further evaluate the proposed framework on additional C-MAPSS subsets (e.g., FD002–FD004) and other degradation datasets with multiple operating conditions and fault modes to comprehensively assess its generalization capability.

Although the proposed model already demonstrates relatively high computational efficiency, further reducing model complexity remains important for deployment in resource-constrained industrial scenarios, such as edge computing devices or embedded monitoring systems. From a structural perspective, the Missing-Parameter Generator and the RUL Predictor have clearly delineated functional roles, enabling potential module-level pruning and simplification. Moreover, in scenarios where sensor failures are infrequent or missing patterns are relatively stable, the missing-parameter generator can be activated on demand and triggered only upon the detection of seriously anomalies or missing data, thereby further reducing average computational overhead. From a sequence modeling standpoint, the current framework employs LSTM to capture temporal dependencies, which may be replaced with lighter temporal modeling structures or combined with temporal window truncation in extremely resource limited deployment environments. Such architectural substitutions preserve the overall generative-predictive integration paradigm and offer substantial engineering flexibility.

Future research work will focus on improving the adaptability and scalability of the framework, especially the performance in challenging scenarios such as facing unknown failure modes and generalization across operating conditions. At the same time, we will also explore how to integrate physical knowledge with data-driven methods more deeply, build more reliable and interpretable prediction models, and provide more complete technical solutions for intelligent operation and maintenance of complex industrial systems.

## Supporting information

S1 Fig Pseudocode of the three-stage training procedure of the proposed generative–regression framework. (TIF)
