# Correction: A deep learning framework for remaining useful life prediction of turbofan engines with partial sensor failure

**DOI:** 10.1371/journal.pone.0357561

**Published:** 2026-09-01

**Authors:** Dongdong Tang

The Funding statement for this article is incorrect. The correct Funding statement is as follows: This work was supported by the Fundamental Research Funds for the Central Universities (Grant No. 25CAFUC03114). The funder had no role in study design, data collection and analysis, decision to publish, or preparation of the manuscript.
