# Supplementary figures and images for: A deep learning framework for remaining useful life prediction of turbofan engines with partial sensor failure
Source: PLoS One. 2026 Apr 29;21(4):e0347312. doi: 10.1371/journal.pone.0347312 (PMC13127949; doi:10.1371/journal.pone.0347312)

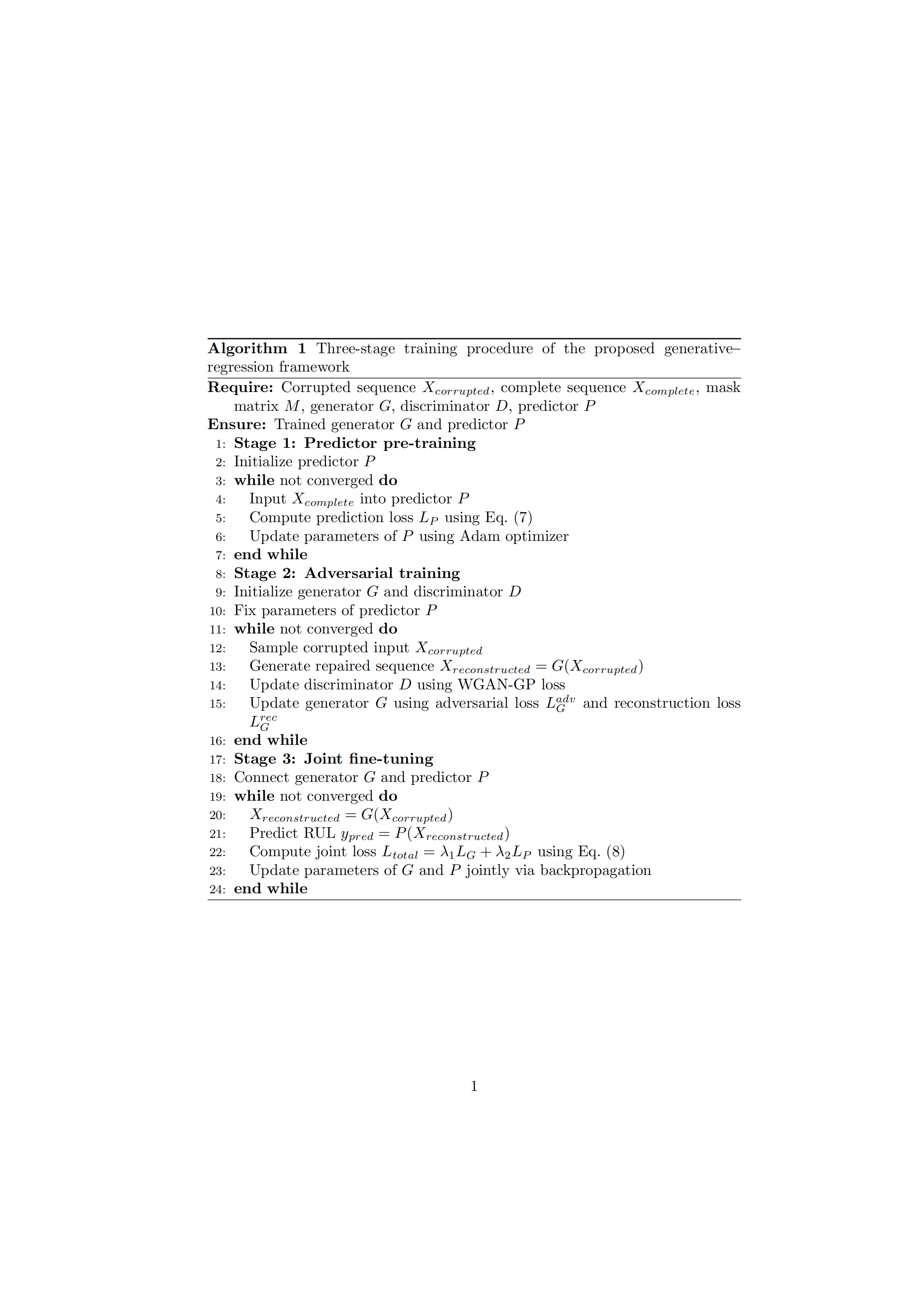

Supplement: S1 Fig — (TIF) [file pone.0347312.s001.tif]
